# Hydrogen Sulfide Suppresses RANKL-Induced Osteoclast Differentiation by Modulating Nrf2/GPX4 Signaling

**DOI:** 10.33549/physiolres.935851

**Published:** 2026-08-01

**Authors:** Zelin HUANG, Ming LAI, Qinglan WEI, Qi CAO, Kang CHEN, Jie ZHANG

**Affiliations:** 1 Department of Pediatric Orthopedics and Hand-Foot Surgery, The Second Affiliated Hospital of University of South China, Hengyang City, China; 2 Department of Joint Surgery, Zhejiang Provincial Veterans Hospital, Jiaxing City, China; 3 Department of Spine Surgery, The Second Affiliated Hospital of University of South China, Hengyang City, China; 4 Hengyang Medical School, University of South China, Hengyang City, China

**Keywords:** Hydrogen sulfide, Osteoclast differentiation, RANKL, Nrf2/GPX4 signaling, Oxidative stress

## Abstract

Excessive osteoclast activity drives bone-resorptive disorders, and oxidative stress is a key regulator of osteoclast differentiation. Hydrogen sulfide (H_2_S), an endogenous antioxidant, may modulate this process, but its mechanisms remain unclear. In this study, RANKL (50 ng/mL) was used to induce osteoclastogenesis in RAW264.7 cells, with or without the H_2_S donor sodium hydrosulfide (NaHS; 50–200 μM). Osteoclast formation was evaluated by TRAP staining and NFATc1/Cathepsin K expression, while oxidative stress, lipid peroxidation, mitochondrial function, and ferroptosis-related parameters were assessed. Mechanistic studies revealed that NaHS promoted Nrf2 nuclear translocation and upregulated GPX4, HO-1, and NQO1, whereas inhibition of Nrf2 or GPX4 partially reversed these effects. NaHS significantly suppressed osteoclast differentiation, alleviated oxidative stress, and restored mitochondrial function. These findings demonstrate that H_2_S inhibits RANKL-induced osteoclastogenesis via the Nrf2/GPX4 pathway and suggest this redox axis as a potential therapeutic target for bone-resorptive diseases.

## Introduction

Bone homeostasis is preserved by a balanced interplay between osteoblast-mediated bone formation and osteoclast-driven bone resorption. Disturbance of this balance—especially heightened osteoclast activity—drives the pathogenesis of disorders such as osteoporosis, rheumatoid arthritis, and osteolytic bone metastasis [1]. Osteoclasts arise from precursors of the monocyte–macrophage lineage, and their differentiation is chiefly regulated by RANKL (receptor activator of nuclear factor-κB ligand) [2]. Binding of RANKL to its receptor RANK triggers multiple downstream cascades, such as NF-κB, MAPK, and NFATc1 signaling, which ultimately promote osteoclast-specific gene expression and the acquisition of bone-resorbing activity [3,4]. Given the central role of RANKL signaling in osteoclastogenesis, elucidating its regulatory mechanisms is of considerable significance for developing therapeutic strategies against bone-resorptive disorders.

Growing evidence suggests that oxidative stress is critically involved in the regulation of RANKL-driven osteoclast differentiation [5,6]. RANKL stimulation markedly elevates intracellular ROS levels, which function not only as secondary messengers to amplify NF-κB and NFATc1 signaling but also as regulators of redox-sensitive molecular events governing osteoclast maturation [7]. Nrf2 (nuclear factor erythroid 2–related factor 2) functions as a central regulator of the cellular antioxidant response. Upon oxidative challenge, it translocates into the nucleus and induces the expression of various cytoprotective genes, thereby contributing to the restoration of redox homeostasis [8]. Among these effectors, glutathione peroxidase 4 (GPX4) is essential for eliminating lipid peroxides and maintaining membrane stability [9]. Although GPX4 is widely recognized as a key suppressor of ferroptosis, emerging evidence suggests that in the context of osteoclastogenesis, GPX4 may primarily function to restrain lipid peroxidation and modulate the oxidative microenvironment, thereby influencing osteoclast formation [10]. Collectively, the Nrf2/GPX4 signaling axis may serve as a key molecular link connecting oxidative stress with osteoclast differentiation.

Hydrogen sulfide (H_2_S), recognized as the third endogenous gasotransmitter following nitric oxide and carbon monoxide, exhibits a wide range of biological functions across cardiovascular, neurological, and skeletal systems [11]. In experimental studies, sodium hydrosulfide (NaHS), a rapid H_2_S donor that dissociates in aqueous solution to generate biologically active H_2_S/HS^−^ species, is widely used to investigate H_2_S-mediated cellular responses [12,13]. Increasing studies have demonstrated that H_2_S possesses potent antioxidant, anti-inflammatory, and cytoprotective properties, largely attributed to its ability to modulate intracellular redox status [14,15]. Within the context of bone metabolism, H_2_S is known to regulate the activities of both osteoblasts and osteoclasts, yet the detailed molecular basis of its actions remains to be fully elucidated [16]. Importantly, H_2_S has been demonstrated to activate Nrf2 and upregulate antioxidant gene expression, thereby limiting ROS accumulation [17]. Whether H_2_S attenuates RANKL-induced osteoclastogenesis through modulation of the Nrf2/GPX4 signaling axis, however, has not been systematically investigated.

Building on these insights, this study explored the impact of H_2_S on RANKL-induced osteoclast differentiation, with particular emphasis on its modulation of oxidative stress and lipid peroxidation via the Nrf2/GPX4 signaling pathway. Using *in vitro* osteoclast precursor models alongside mechanistic validation experiments, we aimed to determine whether Nrf2/GPX4 activation mediates H_2_S’s inhibitory impact on osteoclast formation. Clarifying this mechanism could offer new understanding of redox-dependent osteoclast regulation and reveal potential therapeutic targets for bone-resorptive disorders.

## Materials and Methods

### Cell culture and treatment

RAW264.7 cells (ATCC® TIB-71™) were maintained in DMEM (Gibco, Thermo Fisher Scientific) containing 10 % FBS (Gibco) and 1 % penicillin–streptomycin, and incubated at 37 °C with 5 % CO_2_. For osteoclast differentiation, cells (2 × 10^4^ cells/well, 24-well plates) were treated with recombinant mouse RANKL (50 ng/mL; PeproTech) for 5–7 days, with medium renewal every 48 h [18]. NaHS (Sigma-Aldrich) served as the H_2_S donor. Following preliminary dose screening, NaHS was applied at 50 μM (L-NaHS), 100 μM (M-NaHS), and 200 μM (H-NaHS). NaHS was administered 1 h prior to RANKL stimulation and maintained throughout the differentiation process. For mechanistic inhibition experiments, the Nrf2 inhibitor ML385 (MedChemExpress, Cat# HY-100523) and the GPX4 inhibitor RSL3 (MedChemExpress, Cat# HY-100218A) were added 1 h prior to NaHS treatment at optimized concentrations.

### CCK-8 assay

RAW264.7 cell viability was determined using the Cell Counting Kit-8 (CCK-8, MedChemExpress) following the provided instructions. Cells from each experimental group were seeded into 96-well plates and cultured for 72 h, after which 10 μL of CCK-8 reagent was added to each well and incubated at 37 °C for 1–3 h. Absorbance at 450 nm was measured with a microplate reader, and results were normalized to the control group.

### Tartrate-Resistant Acid Phosphatase (TRAP) staining

Osteoclast formation was analyzed using TRAP staining. After 5–7 days of RANKL exposure, with or without NaHS, cells were fixed in 4 % paraformaldehyde for 15 minutes, washed with PBS, and processed for staining using the TRAP kit (TY-T001-1; Tengyi Biotechnology) following the manufacturer’s guidelines. TRAP-positive multinucleated cells, defined as those with three or more nuclei, were observed and counted using a 40× inverted light microscope. Counts from five randomly selected fields per well were averaged for statistical analysis.

### Flow cytometry analysis of intracellular ROS and lipid peroxidation

Intracellular total ROS and lipid ROS levels were simultaneously detected using DCFH-DA and C11-BODIPY 581/591, respectively. RAW264.7 cells were incubated with 10 μM DCFH-DA and 2 μM C11-BODIPY for 30 min at 37 °C in the dark, followed by three PBS washes, and analyzed by flow cytometry. Mean fluorescence intensity (MFI) values for both probes were normalized against the control group. Decreased or increased MFI values indicated changes in overall oxidative stress and lipid peroxidation levels.

### JC-1 staining

Mitochondrial membrane potential (ΔΨm) was measured using JC-1 dye (Thermo Fisher Scientific, Cat# T3168) following the manufacturer’s guidelines. After the experimental treatments, cells were washed with PBS and incubated with 5 μM JC-1 for 30 min at 37 °C in the dark. Excess dye was removed by PBS washes, and fluorescence was observed using a microscope. Healthy mitochondria with high ΔΨm showed red JC-1 aggregates, while depolarized mitochondria exhibited green monomers. The ΔΨm was quantified by calculating the red-to-green fluorescence ratio with ImageJ software, where a lower ratio reflects mitochondrial depolarization.

### Cellular ATP, MDA, and Fe^2+^ quantification

Levels of ATP, malondialdehyde (MDA), and ferrous iron (Fe^2+^) in cell lysates were determined using commercial assay kits according to the manufacturers’ protocols (ATP: Promega ATP Assay Kit; MDA: Dojindo M496-10; Fe^2+^: FerroOrange). Following cell lysis, these analytes were quantified with a microplate reader. The data were adjusted for total protein levels and presented as nmol/mg protein to enable comparison across different groups.

### Immunofluorescence assay

To examine Nrf2 localization, cells were first fixed with 4 % paraformaldehyde, permeabilized using 0.3 % Triton X-100, and blocked in 5 % BSA. They were then incubated overnight at 4 °C with a rabbit anti-Nrf2 primary antibody (33123-1-AP, Proteintech, Wuhan, China). After PBS washes, a CoraLite® Plus 555-conjugated goat anti-rabbit IgG secondary antibody (Multi-rAb, RGAR003, Proteintech, Wuhan, China) was applied for 1 h at room temperature in the dark. Cell nuclei were stained with DAPI, and fluorescence signals were captured with a confocal laser scanning microscope. Nrf2 nuclear translocation was quantified by comparing nuclear and cytoplasmic fluorescence intensity.

### Western blot analysis

RAW264.7 cells were lysed to obtain total proteins using RIPA buffer, while nuclear and cytoplasmic proteins were isolated with a dedicated extraction kit, following the protocols provided by the manufacturers. Protein levels were first quantified using a BCA assay. Samples containing 20–30 μg of protein were separated via SDS-PAGE and transferred onto PVDF membranes. Membranes were blocked with 5 % non-fat milk for 1 hour at room temperature before being incubated overnight at 4 °C with primary antibodies targeting Nrf2 (1:1000, A21176, ABclonal), GPX4 (1:1000, A27995, ABclonal), HO-1 (1:1000, A11102, ABclonal), NQO1 (1:1000, A19586, ABclonal), NFATc1 (1:1000, A19597, ABclonal), and Cathepsin K (1:1000, DF6614, Affinity Biosciences). For analysis of nuclear translocation, Lamin B (1:25,000, AC057, ABclonal) was used as a nuclear marker, whereas β-actin (1:5000, AF7018, Affinity Biosciences) served as a loading control for cytoplasmic or total protein. Protein bands were detected using enhanced chemiluminescence following incubation with the corresponding HRP-conjugated secondary antibodies, and their intensity was measured through densitometry.

### Statistical analysis

Each experiment was repeated at least three times independently (n ≥ 3), and results are shown as mean ± standard deviation (SD). For multiple-group comparisons, one-way ANOVA with Tukey’s post hoc test was applied, while two-group comparisons were analyzed using a two-tailed Student’s t-test. Statistical analyses were carried out using GraphPad Prism 8.0, with *p* < 0.05 considered statistically significant.

## Results

### H_2_S suppresses RANKL-induced differentiation of osteoclasts in RAW264.7 cells

**Fig. 1:**
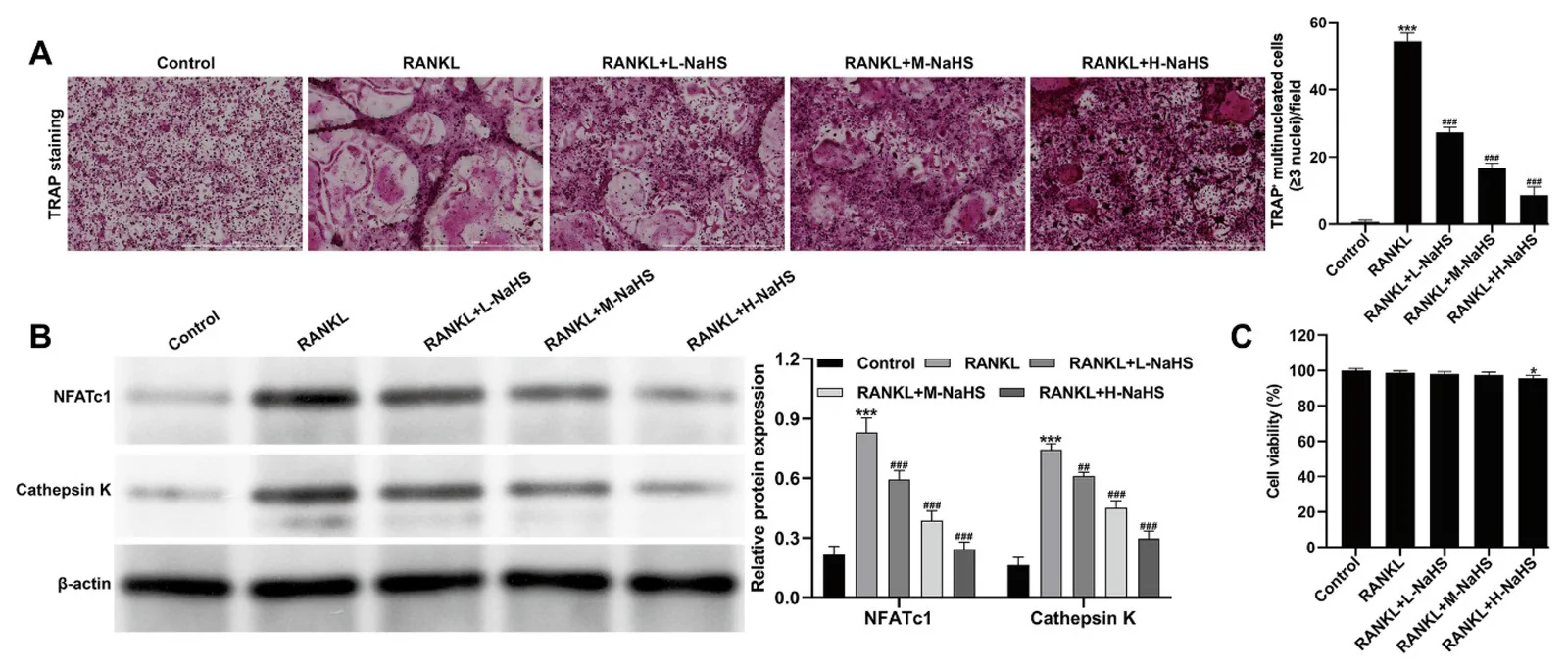
H_2_S suppresses RANKL-induced differentiation of osteoclasts in RAW264.7 cells. (**A**) Representative images of TRAP staining and quantitative analysis of TRAP-positive multinucleated cells (≥3 nuclei) in RAW264.7 cells treated with RANKL (50 ng/mL) in the presence or absence of NaHS (L-, M-, or H-NaHS) for 5–7 days. Scale bar = 1000 μm. (**B**) Western blot analysis of osteoclastogenic markers NFATc1 and Cathepsin K in RAW264.7 cells treated as indicated. (**C**) Cell viability of RAW264.7 cells treated with NaHS (L-, M-, or H-NaHS) for 72 h, assessed by CCK-8 assay. Data are presented as mean ± SD from at least three independent experiments. Statistical analysis was performed using one-way ANOVA followed by Tukey’s post hoc test. ***p < 0.001, *vs*. control; ##*p* < 0.01, *vs*. RANKL; ###*p* < 0.001, *vs*. RANKL

To investigate whether H_2_S affects RANKL-induced osteoclastogenesis, RAW264.7 cells were treated with RANKL with or without NaHS (L-, M-, or H-NaHS). RANKL strongly promoted osteoclast differentiation, as indicated by a pronounced increase in TRAP-positive multinucleated cells compared with untreated controls (Fig. 1A). NaHS treatment inhibited osteoclast formation in a dose-dependent fashion, with L-NaHS showing modest inhibition, M-NaHS producing pronounced suppression, and H-NaHS exhibiting the strongest effect (Fig. 1A). Western blot analysis confirmed that RANKL robustly upregulated the osteoclastogenic transcription factor NFATc1 and its downstream effector Cathepsin K, whereas NaHS dose-dependently attenuated their expression (Fig. 1B), supporting the molecular basis for H_2_S-mediated inhibition of osteoclast differentiation. Although H-NaHS produced maximal inhibition, CCK-8 assays revealed a slight decrease in RAW264.7 cell viability, while L-NaHS and M-NaHS did not significantly affect viability (Fig. 1C). Considering both efficacy and cellular safety, M-NaHS was selected for all subsequent mechanistic studies.

### H_2_S suppresses RANKL-induced oxidative stress and ferroptosis-like mitochondrial dysfunction

**Fig. 2:**
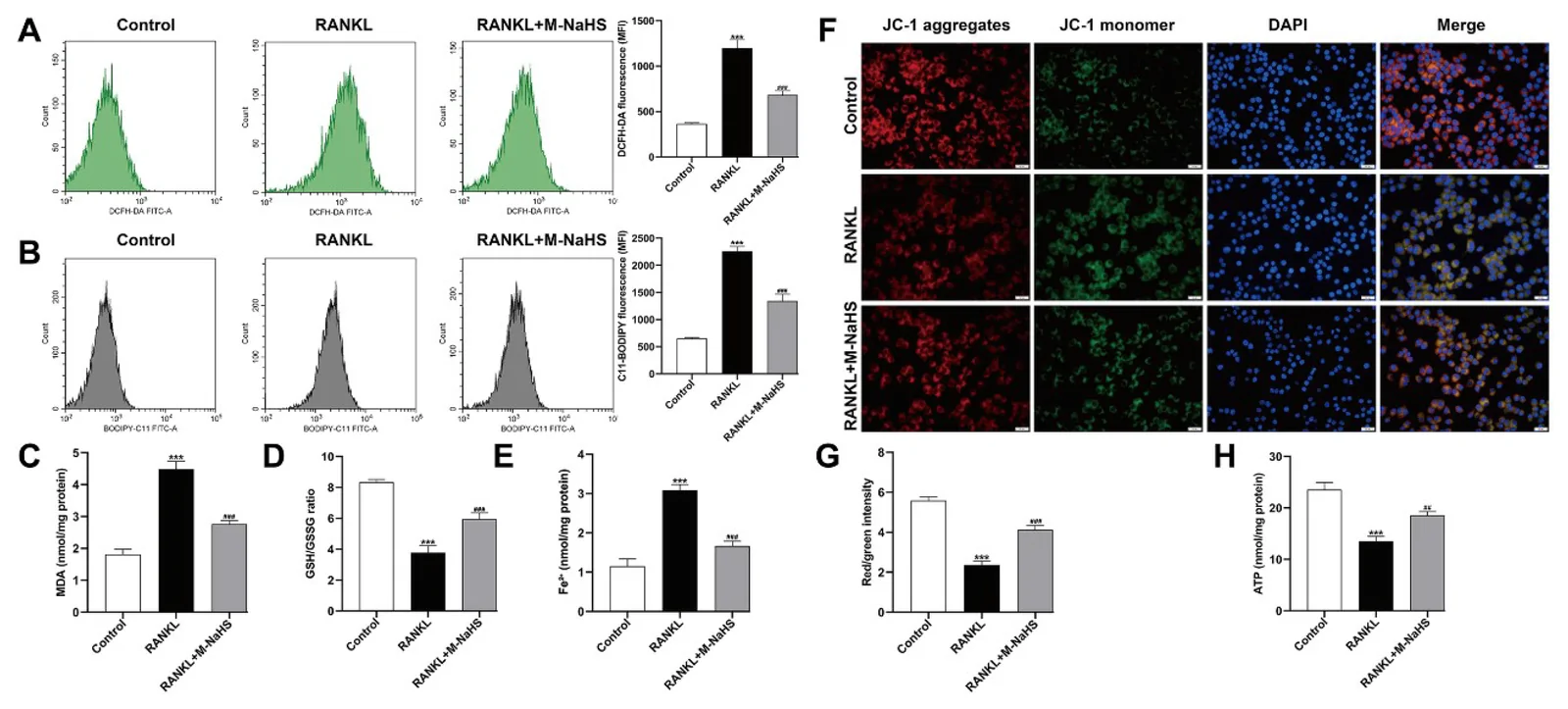
H_2_S suppresses RANKL-induced oxidative stress and ferroptosis-like mitochondrial dysfunction. (**A**) Flow cytometric analysis of intracellular ROS (DCFH-DA) in RAW264.7 cells treated with RANKL (50 ng/mL) with or without M-NaHS for 72 h. (**B**) Flow cytometric analysis of lipid peroxidation (C11-BODIPY) under the indicated conditions. (**C**) Malondialdehyde (MDA) content in nmol/mg protein. (**D**) Cellular GSH/GSSG ratio, reflecting antioxidant capacity. (**E**) Intracellular Fe^2+^ levels (mmol/mg) indicating ferroptosis-like changes. (**F–G**) Mitochondrial membrane potential assessed by JC-1 red/green fluorescence ratio. (**H**) Cellular ATP content (nmol/mg protein). Data are presented as mean ± SD from at least three independent experiments. Statistical analysis was performed using one-way ANOVA followed by Tukey’s post hoc test. ***p < 0.001, *vs*. control; ##*p* < 0.01, ###*p* < 0.001, *vs*. RANKL

Given that oxidative stress contributes to RANKL-induced osteoclast differentiation, we next evaluated whether H_2_S modulates ROS production, lipid peroxidation, and mitochondrial function in RAW264.7 cells. Flow cytometric analysis revealed that RANKL markedly increased intracellular ROS levels, as indicated by DCFH-DA fluorescence, whereas M-NaHS treatment significantly reduced ROS accumulation, restoring levels close to those of control cells (Fig. 2A). Consistently, lipid peroxidation, assessed by C11-BODIPY oxidation, was elevated following RANKL stimulation but effectively attenuated by H_2_S (Fig. 2B). Biochemical assays further confirmed these effects: RANKL induced a substantial increase in MDA content and a concomitant decrease in the GSH/GSSG ratio, indicative of heightened oxidative stress and impaired antioxidant capacity, both of which were reversed by H_2_S treatment (Fig. 2C–D). In addition, intracellular Fe^2+^ levels were elevated upon RANKL stimulation, reflecting ferroptosis-like changes, and were significantly reduced by H_2_S (Fig. 2E). Mitochondrial function was similarly impacted: RANKL reduced the JC-1 red/green fluorescence ratio, reflecting a decline in mitochondrial membrane potential (Fig. 2F–G), and reduced cellular ATP levels. Treatment with H_2_S restored ΔΨm and ATP content toward control levels (Fig. 2H).

### H_2_S modulates Nrf2/GPX4 antioxidant signaling in RANKL-treated osteoclasts

**Fig. 3:**
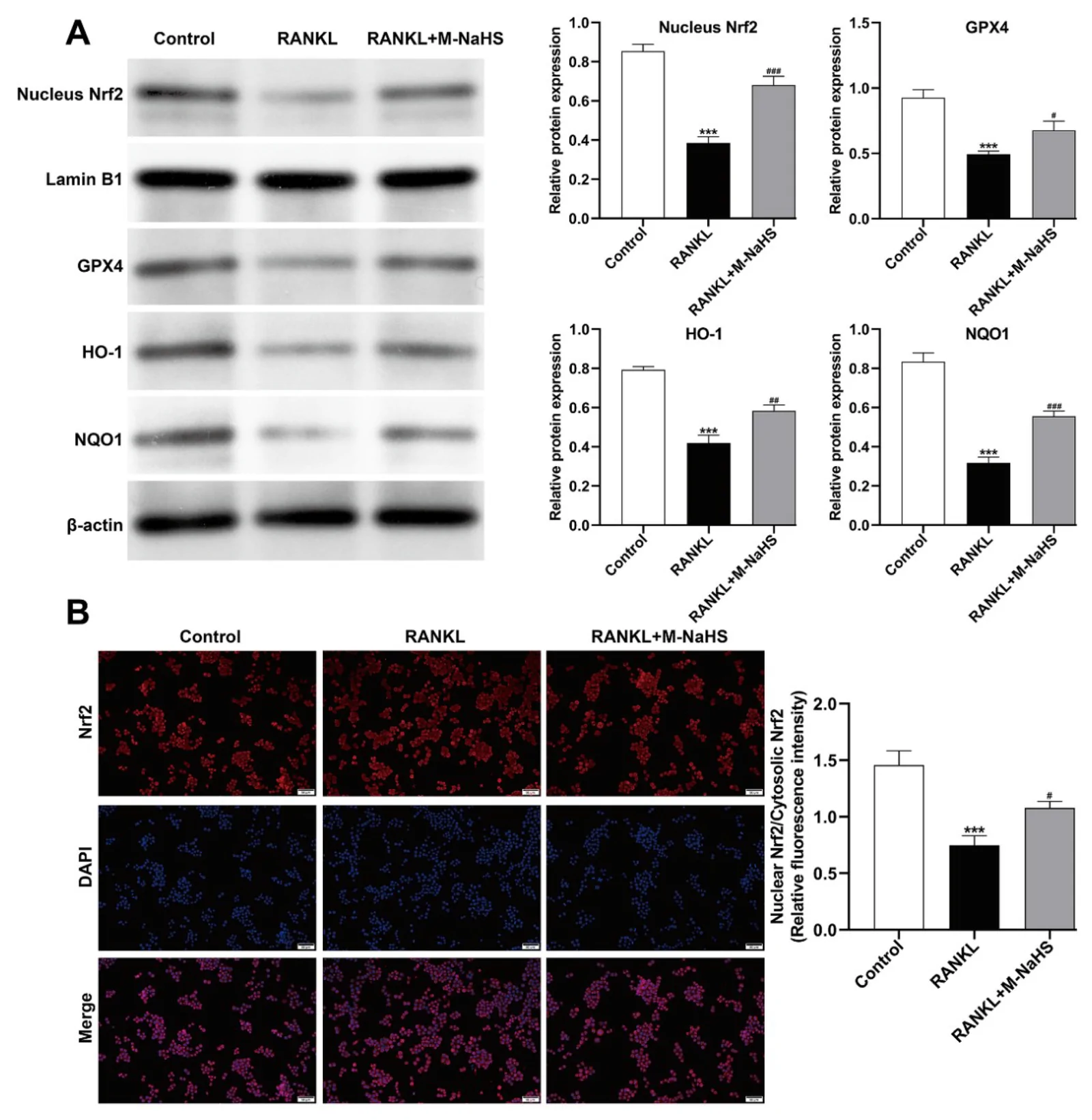
H_2_S modulates Nrf2/GPX4 antioxidant signaling in RANKL-treated osteoclasts. (**A**) Western blot analysis of nuclear Nrf2, GPX4, HO-1, and NQO1 in RAW264.7 cells treated with RANKL (50 ng/mL) in the presence or absence of M-NaHS for 72 h. Lamin B and β-actin were used as nuclear and total protein loading controls, respectively. (**B**) Immunofluorescence analysis showing nuclear and cytoplasmic Nrf2 in RAW264.7 cells under the indicated conditions. Quantification of the nuclear/cytoplasmic Nrf2 fluorescence intensity ratio is shown. Scale bar = 20 μm. Data are presented as mean ± SD from at least three independent experiments. Statistical analysis was performed using one-way ANOVA followed by Tukey’s post hoc test. ***p < 0.001, *vs*. control; #*p* < 0.05, ##*p* < 0.01, ###*p* < 0.001, *vs*. RANKL.

To explore whether H_2_S protects against RANKL-induced oxidative stress through the Nrf2/GPX4 antioxidant pathway, RAW264.7 cells were exposed to RANKL with or without M-NaHS treatment. Western blot analysis revealed that RANKL treatment significantly lowered nuclear Nrf2 levels and downregulated GPX4, HO-1, and NQO1 expression, suggesting weakened antioxidant defenses and increased oxidative stress (Fig. 3A). H_2_S treatment significantly enhanced Nrf2 translocation to the nucleus and restored the levels of GPX4, HO-1, and NQO1, indicating activation of the Nrf2/GPX4-mediated antioxidant pathway. Consistently, immunofluorescence analysis demonstrated that H_2_S markedly enhanced Nrf2 nuclear localization compared with RANKL treatment alone, as reflected by an increased nuclear-to-cytoplasmic Nrf2 ratio (Fig. 3B), further confirming activation of Nrf2 signaling.

### The Nrf2/GPX4 pathway underlies H_2_S’s protective effect against RANKL-induced osteoclast differentiation

**Fig. 4:**
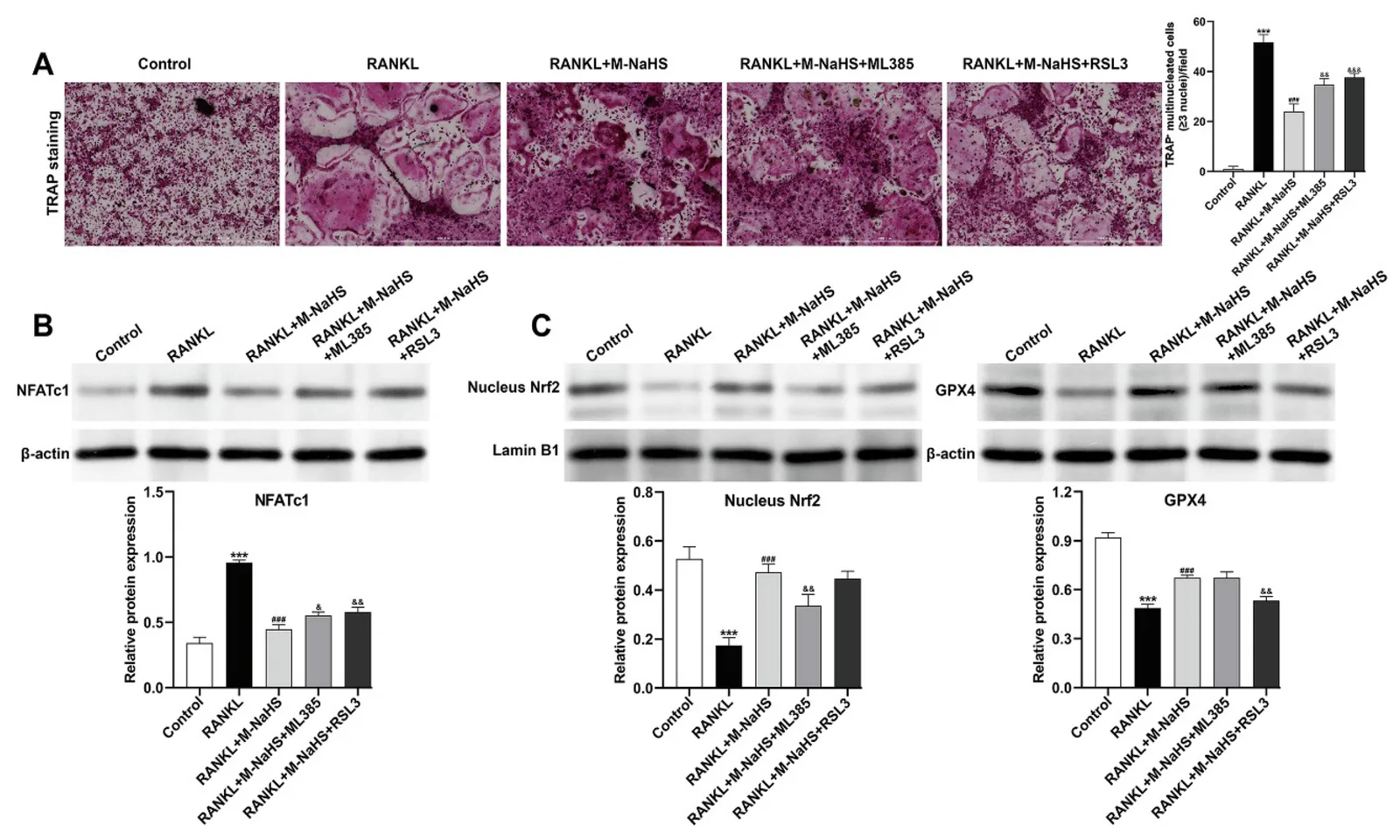
The Nrf2/GPX4 pathway underlies H_2_S’s protective effect against RANKL-induced osteoclast differentiation. (**A**) Representative images of TRAP staining and quantification of TRAP-positive multinucleated cells (≥3 nuclei) in RAW264.7 cells treated with RANKL (50 ng/mL) ± M-NaHS, with or without ML385 or RSL3 for 5–7 days. Scale bar = 1000 μm. (**B**) Western blot analysis of NFATc1 in RAW264.7 cells under the indicated conditions. (**C**) Western blot analysis of nuclear Nrf2 and GPX4 in RAW264.7 cells treated as indicated. Data are presented as mean ± SD from at least three independent experiments. Statistical analysis was performed using one-way ANOVA followed by Tukey’s post hoc test. ***p < 0.001, *vs*. control; ###*p* < 0.001, *vs*. RANKL; &*p* < 0.05, &&*p* < 0.01, &&&*p* < 0.001, *vs*. RANKL+ M-NaHS

To assess whether H_2_S suppresses osteoclastogenesis through Nrf2/GPX4 signaling, RAW264.7 cells were exposed to RANKL with or without M-NaHS, in the presence or absence of the Nrf2 inhibitor ML385 or the GPX4 inhibitor RSL3. TRAP staining showed that H_2_S strongly inhibited the generation of RANKL-induced multinucleated osteoclasts, while simultaneous treatment with ML385 or RSL3 partially reversed this suppression of osteoclast differentiation (Fig. 4A). Consistently, RANKL stimulation reduced nuclear Nrf2 and GPX4 protein levels while markedly increasing the expression of the osteoclastogenic transcription factor NFATc1 (Fig.s 4B–C). In contrast, inhibition of Nrf2 or GPX4 partially reversed these effects, with NFATc1 expression rising and Nrf2/GPX4 activation attenuated.

### H_2_S mitigates RANKL-induced oxidative stress and ferroptosis by activating the Nrf2/GPX4 signaling pathway

**Fig. 5:**
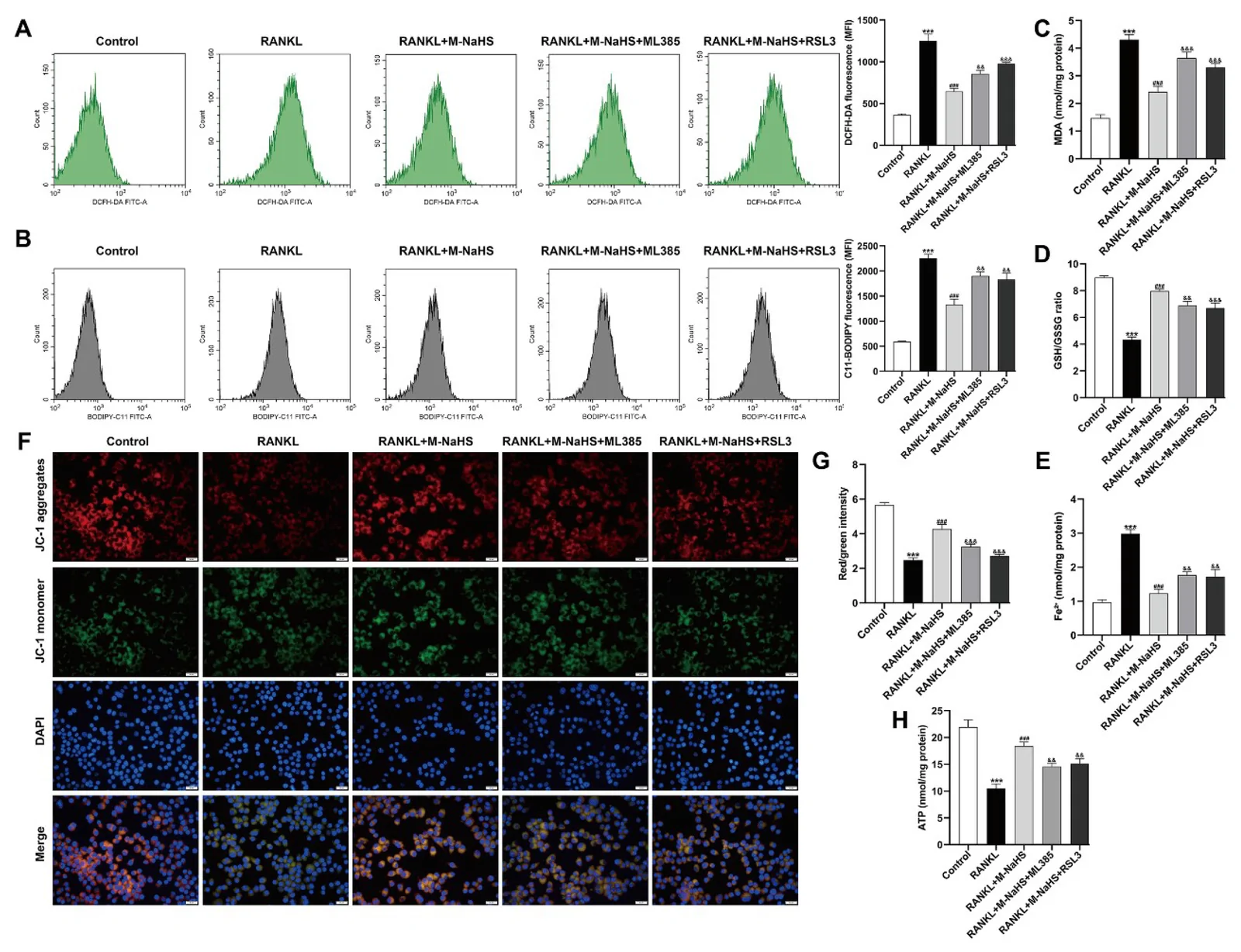
H_2_S protects against RANKL-induced oxidative stress and ferroptosis through activation of the Nrf2/GPX4 pathway. RAW264.7 cells were treated with RANKL (50 ng/mL) ± M-NaHS, with or without ML385 (Nrf2 inhibitor), or RSL3 (GPX4 inhibitor) for 5–7 days. (**A**) Flow cytometric analysis of intracellular ROS levels using DCFH-DA. (**B**) Flow cytometric assessment of lipid peroxidation with C11-BODIPY 581/591. (**C**) Cellular MDA content. (**D**) GSH/GSSG ratio as a measure of antioxidant capacity. (**E**) Intracellular Fe^2+^ levels. (**F–G**) Mitochondrial membrane potential (ΔΨm) evaluated by JC-1 staining; red/green fluorescence ratio shown. (**H**) Cellular ATP levels. Data are presented as mean ± SD from at least three independent experiments. Statistical analysis was performed using one-way ANOVA followed by Tukey’s post hoc test. ***p < 0.001, *vs*. control; ##*p* < 0.01, ###*p* < 0.001, *vs*. RANKL; &&*p* < 0.01, &&&*p* < 0.001, *vs*. RANKL+ M-NaHS

We next investigated whether the antioxidant and mitochondria-protective effects of H_2_S were mediated by Nrf2/GPX4 signaling. Flow cytometric analysis revealed that RANKL markedly elevated intracellular ROS levels (Fig. 5A) and lipid peroxidation (Fig. 5B) relative to control cells. H_2_S treatment significantly reduced both total ROS and lipid peroxidation toward control levels. Co-treatment with ML385 or RSL3 partially abrogated the effect of H_2_S, resulting in higher ROS and lipid peroxidation than H_2_S alone. Consistent with these findings, RANKL induced a pronounced increase in MDA content (Fig. 5C) and Fe^2+^ accumulation (Fig. 5E), accompanied by a decreased GSH/GSSG ratio (Fig. 5D), indicating enhanced oxidative stress and ferroptosis-like changes. H_2_S restored these parameters to near baseline, whereas ML385 or RSL3 co-treatment partially reversed these protective effects. Mitochondrial membrane potential was evaluated using the JC-1 red-to-green fluorescence ratio (Fig. 5F–G), was significantly reduced by RANKL, indicating ΔΨm loss. H_2_S restored ΔΨm, whereas ML385 or RSL3 co-treatment attenuated this rescue. Similarly, RANKL decreased cellular ATP content (Fig. 5H), which was recovered by H_2_S and partially reversed by Nrf2 or GPX4 inhibition. Together, these findings suggest that Nrf2/GPX4 mediates H_2_S protection against RANKL-induced oxidative stress and ferroptosis.

## Discussion

Excessive osteoclast differentiation and activation are fundamental drivers of bone-destructive diseases, including rheumatoid arthritis, osteoporosis, and tumor-associated osteolysis [19,20]. Osteoclastogenesis is orchestrated by RANKL-dependent signaling cascades that converge on NFATc1, the master transcription factor governing osteoclast-specific gene expression [21]. Beyond canonical inflammatory pathways, redox signaling has emerged as a critical modulatory layer in osteoclast differentiation [5,22]. RANKL-induced ROS production functions not merely as a byproduct of metabolic activation but as a signaling amplifier that enhances NF-κB and NFATc1 activity [5]. Thus, osteoclastogenesis can be viewed as a redox-sensitive differentiation program, underscoring the importance of identifying antioxidant regulatory nodes capable of restraining pathological bone resorption.

H_2_S is now widely recognized as a gaseous messenger with significant roles in antioxidant defense and cellular protection [23]. Using RAW264.7 cells, a well-established model of RANKL-induced osteoclastogenesis [24], our results showed that NaHS inhibits osteoclast differentiation in a dose-dependent way, with moderate concentrations achieving optimal efficacy without cytotoxicity, consistent with previous observations regarding H_2_S donor pharmacodynamics [25]. Our findings align with earlier work showing that NaHS inhibits human CD11b^+^ osteoclast progenitor differentiation through NRF2-dependent antioxidant activation [26]. However, while prior research primarily established NRF2 as an upstream antioxidant regulator [26], our data extend this paradigm by identifying the Nrf2/GPX4 axis as a functional effector module that links redox regulation to ferroptosis-like mitochondrial integrity. This distinction is mechanistically significant, as it shifts the conceptual framework from general antioxidant activation to a specific lipid peroxidation–detoxifying pathway that directly constrains osteoclastogenic signaling. It should be noted, however, that the effects of H_2_S on osteoclast biology appear to be context-dependent. While our findings demonstrate an inhibitory effect of H_2_S on RANKL-induced osteoclastogenesis, previous studies have reported that H_2_S may promote osteoclast differentiation through activation of the PI3K/AKT/mTOR pathway under certain experimental conditions [27]. These discrepancies may be attributable to differences in cell type, H_2_S donor characteristics, concentration, exposure duration, and the specific signaling networks examined. Collectively, the available evidence suggests that H_2_S functions as a pleiotropic regulator of osteoclastogenesis, with its biological effects likely determined by the cellular redox state and microenvironmental context.

Oxidative stress is a recognized promoter of osteoclast differentiation [5,22], while Nrf2 acts as a principal regulator of cellular antioxidant defenses, modulating the expression of genes like NQO1, HO-1, and GPX4 [28]. GPX4 is uniquely positioned at the intersection of redox biology and ferroptosis, as it detoxifies lipid hydroperoxides and prevents iron-dependent membrane damage [29,30]. Emerging studies suggest that ferroptosis-related lipid peroxidation may influence bone remodeling and osteoclast function [31], although its precise mechanistic contribution remains incompletely defined. Our results indicate that RANKL induces a ferroptosis-like phenotype characterized by ROS accumulation, lipid peroxidation, Fe^2+^ overload, mitochondrial membrane potential collapse, and ATP depletion. Importantly, H_2_S restored redox equilibrium and mitochondrial function via Nrf2/GPX4 activation, while pharmacological inhibition of Nrf2 or GPX4 partially reversed these effects. These findings support a model in which RANKL-driven osteoclastogenesis is coupled to a lipid peroxidation–dependent metabolic shift, and H_2_S interrupts this process by reinforcing GPX4-mediated membrane protection. Thus, Nrf2/GPX4 may function as a redox checkpoint that constrains excessive osteoclast differentiation under oxidative stress conditions.

Several limitations merit consideration. First, the present study is confined to an *in vitro* RAW264.7 model, and *in vivo* validation in osteolytic disease models will be required to confirm translational relevance. Second, although ML385 and RSL3 were employed to interrogate pathway dependence [32,33], genetic manipulation of Nrf2 or GPX4 would provide more definitive mechanistic validation. Third, NaHS was used as a pharmacological H_2_S donor in the present study. Although widely employed in H_2_S research, its release kinetics may not fully recapitulate the spatial and temporal regulation of endogenous H_2_S production. Therefore, future studies using slow-releasing H_2_S donors or modulation of endogenous H_2_S-generating enzymes are warranted to further validate the physiological relevance of our findings. Fourth, while we identified ferroptosis-like biochemical alterations, comprehensive ultrastructural and lipidomic characterization remains to be performed. Future studies should also explore potential crosstalk between Nrf2/GPX4 signaling and canonical osteoclastogenic pathways, including NF-κB and MAPK, to fully delineate the regulatory network architecture.

Collectively, our results support a model in which RANKL-induced osteoclast differentiation is driven by redox imbalance and lipid peroxidation, forming a feed-forward loop that amplifies osteoclastogenic signaling. H_2_S acts as a redox rheostat, restoring oxidative balance via Nrf2/GPX4 activation, limiting ferroptosis-like mitochondrial dysfunction, and suppressing excessive osteoclast maturation. By highlighting GPX4-dependent lipid peroxide detoxification downstream of NRF2, our study establishes the Nrf2/GPX4 axis as a critical oxidative checkpoint in osteoclast biology and suggests that modulating H_2_S or ferroptosis pathways may offer therapeutic potential in bone-resorptive diseases.
